# Participatory System Mapping of a Hospice Care System With Hospice Stakeholders: Hybrid Design Workshop Study

**DOI:** 10.2196/69746

**Published:** 2026-07-24

**Authors:** Andrew Tibbles, Farnaz Nickpour

**Affiliations:** 1 School of Engineering, University of Liverpool, The Quadrangle, Brownlow Hill, Liverpool, L69 3GH, United Kingdom

**Keywords:** palliative and end-of-life care, participatory system mapping, design workshops, stakeholder engagement, participatory research methods

## Abstract

**Background:**

Palliative and end-of-life care (PEoLC) systems are expanding across services, settings, and stakeholders, increasing their complexity and the need for systemic understanding to support patient outcomes and service delivery. Hospice care is central to the future of PEoLC, as hospices provide holistic services and engage diverse stakeholders. Participatory system mapping offers a way to collectively understand and visualize complex dynamics with those who live and work within these systems.

**Objective:**

This study aims to capture hospice system dynamics and preliminary leverage points via participatory causal loop diagram (CLD) mapping while evaluating method suitability through 3 research questions: (RQ1) What key variables and causal interrelationships do stakeholders identify in a hospice through participatory system mapping workshops? (RQ2) What preliminary leverage points emerge from the system map? and (RQ3) How effective are participatory system mapping workshops for capturing hospice dynamics?

**Methods:**

We developed and iteratively refined an innovative hybrid, asynchronous, multimodal design workshop series in a hospice in North West England. Stakeholders were introduced to core concepts in technology, design, systems thinking, and CLDs before engaging in participatory system mapping focused on the hospice experience quality. CLDs generated in workshops and through asynchronous participation were consolidated into a composite hospice system map. Twenty-seven participants, including patients, health care professionals, volunteers, managers, maintenance staff, and chaplaincy, contributed to the mapping process. The resulting map was analyzed using quantitative network analysis (in-degree, out-degree, betweenness, and closeness centrality) alongside qualitative interpretation of key system dynamics.

**Results:**

The participatory hospice system map contained 84 variables connected by 175 causal links. Network analysis highlighted patient experience (highest in-degree, 20), advanced care planning (highest out-degree, 8), fundraising (highest betweenness centrality, 0.19), and relationships with community organizations and external stakeholders (highest closeness centrality, 0.23) as central elements in the map. Qualitative analysis illuminated important dynamics, including the impact of hospital admissions and hospice stereotypes, as well as uncertainties around how advanced care planning is shaped and enacted in practice.

**Conclusions:**

Participatory system mapping with hospice stakeholders was feasible in a time-pressured setting and generated a nuanced, stakeholder-led representation of hospice system dynamics. The hybrid, multimodal workshop model enhanced access and flexibility, supporting diverse engagement. Network analysis of the CLD suggested preliminary structural and conceptual leverage points and revealed gaps in shared understanding, indicating candidate areas for service development, policy attention, and further research. Future work should examine the replicability of this approach across PEoLC settings and integrate context-specific processes to validate and act on candidate leverage points.

## Introduction

### Hospice as a Research Setting

In England, palliative and end-of-life care (PEoLC) refers to care that enables people of all ages to live well in the last years, months, weeks, days, and hours of life, with “end of life” specifically referring to the final year of life. These terms are often combined to describe the broad services that provide PEoLC [1], which is the term used here. PEoLC systems face intensifying pressures, with aging populations driving demand while services expand multidimensionally to address patient needs [2-6], in-person and digital services [3,7-11], diverse care environments [7,12], its changing societal value [13,14], and a person-centered care approach [15]. PEoLC definitions are evolving through "upstream migration," as services integrate earlier into primary and hospital care rather than being limited solely to end-of-life, expanding the range of illnesses included [16,17]. Hospice care, as a core pillar of PEoLC, reflects this expansion, evolving from single-site cancer relief to multifaceted inpatient and community services within regional networks (Multimedia Appendix 1 summarizes these expansions across dimensions). This expansion and complexity have received recognition from both within and outside the field to approach it on a systemic level [18-22]. Current understanding lags behind these developments, risking reactive services rather than proactive redesign.

Systemic design addresses such complexity with place-based, stakeholder-involved mapping for collective understanding and person-centered interventions [23-25]. Participatory system mapping via causal loop diagrams (CLDs) visualizes this by constructing dynamics for discovery and leverage identification, building joint understanding, and leading to greater trust and sustainability of interventions [26,27]. Distinct from expert group model building (GMB), it engages end users (patients or carers) via directional variable maps for whole-system experience perspectives [28-32]. Participatory system mapping, as a flexible method, is more closely aligned to the format of CLDs, which are directional system maps of variables and their causal relations [33]. By making visible the variables, relationships, and gaps that shape hospice care, participatory CLD mapping can support a more holistic understanding of how services, staff, patients, families, and wider networks interact, thereby identifying points where more coordinated and person-centered care may be strengthened.

Health care participatory design principles and approaches that involve stakeholders are increasing in policy prominence and are seen as enriching the research process, producing better outcomes [34-37]. The National Health Service (NHS) has embedded co-design principles in its “Long Term Plan”; however, they risk tokenism, lack standardization and designers in the process, and are unable to adapt to time or ethical barriers [20,24,25,38,39].

Hospices provide an ideal setting for the exploration of participatory design methods and for researching PEoLC dynamics and operations, compared with alternative locations, by providing access to the core of PEoLC operations and its branching specialist services. This study has selected a hospice operating as a branch of a national hospice care charitable organization in the United Kingdom, which provides robust ground for a holistic, granular, and place-based understanding of hospice care as a complex system. The particular hospice was selected based on several factors, including proximity, willingness for interdisciplinary collaboration, a strong research culture, and support for the researchers in navigating the system and engaging stakeholders.

### Objectives and Research Questions

Our objective is to capture hospice system dynamics and preliminary leverage points via participatory CLD mapping while evaluating method suitability. We have devised 3 research questions in order to achieve this objective: RQ1. What key variables and causal interrelationships do stakeholders identify in a hospice via participatory system mapping workshops? RQ2. What preliminary leverage points emerge from the system map? RQ3. How effective are participatory system mapping workshops in capturing hospice dynamics?

## Methods

### Ethical Considerations

This study formed part of a doctoral research project and received ethical approval from the University of Liverpool, Central University Research Ethics Committee (Ref: 10901; approval document and protocol in Multimedia Appendices 2 and 3). Informed consent was obtained from all participants.

### Participatory System Mapping Approach

Participatory approaches toward system mapping in health care are assessed as overall positive, with methodological challenges and barriers involved [40,41]. Jun et al [41] note the adaptation of action research methods due to the time constraints of stakeholders in time-limited, one-off workshop settings, as well as the recruitment and retention of busy and geographically distributed stakeholders.

Hospice care is treated here as a focal subsystem of PEoLC because it brings together inpatient, community, bereavement, volunteer, and organizational functions, making it a useful microcosm of broader PEoLC system dynamics. Using participatory research methods in this setting introduces advantages and known barriers similar to those in broader health care settings. The advantages include incorporating many voices collaboratively, as well as reflexive and adaptive approaches that can feed into working groups, continuous improvement, and wider service innovation. The barriers include the high cost of implementation; the time required to conduct research and build productive relationships between researchers and participants; ethical considerations around including patients in sensitive topics; and the challenges of initiating and sustaining participation [35,36,41-44]. This focus allows the study to examine how broader PEoLC dynamics are expressed in a service setting where clinical, community, and organizational congregate.

Adopting the recommendations of Hockley et al [42] and Borgstrom and Barclay [44], the researchers attempted to gain managerial buy-in early on, be reflexive and responsive to participants’ needs, and enable equitable engagement during workshops through effective facilitation. The study adopted a participatory system mapping of CLD through an asynchronous workshop format to allow for flexible, enhanced, and inclusive engagement. CLD was selected because its mapping rules were simple, and once learned, a CLD could be easily understood and built upon by other participants in a nonexpert workshop setting.

### Advanced Workshop Design and Development Approach: Developing Participatory, Hybrid Multimodal System Mapping Workshops With Stakeholders

Four consecutive 1-hour workshop sessions were developed. A design approach was adopted, and the participatory system mapping was framed, introduced, and delivered as part of a wider design workshop series for system stakeholders (hospice staff and patients). The content of the workshops aimed to first increase participants’ literacy and confidence in the meaning, potential value, and role of technology, design, and systems in hospice care, and then conduct participatory mapping of the hospice system with participants using a CLD. The wider design-led approach to first engage with and understand the relevance and importance of systems design in future hospice care is argued to build contextual relevance and offer value and meaning to participants, thereby increasing meaningful engagement.

“Hospice experience quality” was selected as the central variable for cocreation of the CLD by workshop participants, as it was considered a relevant and important area of priority for all hospice stakeholders, including patients and staff, due to its direct impact on the overall care and well-being of individuals at the end of life.

Accessible and jargon-free language was used in all content, and any potentially new concepts were explained. An information poster was designed for each workshop, outlining its remit and summarizing its content. The language, accessibility, and functionality of all print and digital material were assessed by a patient-facing staff member with no previous familiarity with the topic or content, and improvements were made accordingly. Enhanced engagement and interaction were encouraged by displaying the information poster from the previous workshop in a public space within the hospice, with the ability for participants to add to the discussion via a large notepad, Post-it notes, and pens. Table 1 provides a summary of the 4 workshops, outlining content, aims, and activities.

**Table 1.** Workshop design—content, aims, and activities.

Workshops	Content and aim	Activity
Workshop 1: technology	Content: The most recent technological advancements selected for their perceived importance to future hospice care (robotics and artificial intelligence) [10] Aim: To consider challenges and opportunities for using technologies in the context of hospice care	Discussing pros, cons, opportunities, and concerns of technologies in hospice care.
Workshop 2: design	Content: The definition of design, an introduction to human-centered design and design thinking principles, and 4 orders of design [45] Aim: To provide a broad perspective of design and a general understanding of the value and role of design in hospice care	Open discussion about what design is, design processes, design connection to PEoLC^a^, and the impact of design on the world, with a neutral stance presenting good and bad examples.
Workshop 3: understanding complexity	Content: An introduction to systemic design, its remit and application, and how to make and read a CLD^b^ Aim: To understand the aim, processes, and applications of systems design and to understand CLD	Filling out a CLD template (reinforcing and balancing loop diagram) individually.
Workshop 4: mapping complexity	Content: A brief reintroduction to CLD. Discussion of CLD outcomes at the end of the workshop Aim: To create a CLD around the variable of “hospice experience quality”	Participatory system mapping using CLD and building a map as a group using paper, pens, and Post-it notes.

a PEoLC: palliative and end-of-life care.

b CLD: causal loop diagrams.

The workshops were conducted over a period of 3 months in a space accessible to staff, patients, and family members within the hospice, facilitated by an experienced design researcher. At the time of running the workshops, the researcher had spent 8 months in the hospice as part of their designer-in-residence role, conducting observations and interviews, and had developed a good level of familiarity with staff and patients.

The workshop format took shape iteratively throughout the research, exploring the most suitable formats in a high-pressure and sensitive health care setting for participants with very limited time and mental space to engage. This required flexibility in a highly responsive, patient-facing workplace with immediate and fluctuating patient care needs.

### Workshop Iteration 1: Asynchronous and In-Person, With an Emphasis on Physical Interactions

The first iteration was informed theoretically by the work of Davis et al [46] and empirically by the format of an existing well-attended 1-hour weekly journal club for hospice staff. Staff engagement helped suggest and later verify a 1-hour duration, a set time slot, and a daily in-person physical format as optimal for attendance and interaction. Four daily 1-hour workshops were delivered consecutively over 1 week, at a consistent time. However, workshop attendance revealed several barriers to participation. Overall, 8 staff members were able to engage over the week, 1 staff member attended all workshops, and no patients participated. Feedback collected from the staff and patients, focused primarily on format and timing, is outlined in Textbox 1.

Textbox 1. **The first iteration of the participatory system mapping workshop—format, barriers, and suggestions.**

**Iteration 1**

- Format
  - Four daily 1-hour workshops delivered consecutively over 1 week, at a consistent time
  - Each workshop had a printed poster summary and an assigned activity, made available to participants unable to attend the live workshop, to allow asynchronous engagement with the content
- Barriers
  - Staff’s inconsistent attendance and nonattendance due to unavoidable reasons (eg, immediate patient care needs, overrunning shifts, or unplanned external visits)
  - Patients’ nonattendance due to fluctuating health and care needs and physical and mental capacity
- Suggestions
  - More accessible and flexible options for workshop interaction were as follows:
    - An online space where audiovisual presentations of information could be easily accessed, paused, and resumed
    - Online spaces where activities could be completed online at the participant’s convenience
    - Options for individual or small-team sessions at flexible times

### Workshop Iteration 2: Asynchronous, Extended, and Multimodal Hybrid, With an Emphasis on Flexible Engagement and Interaction

#### Iterative Refinement of the Workshop Format

Building on the feedback, the workshop format was iterated to include 4 core characteristics around content, modes of activity, advertising, and access:

- Online content: A website was designed containing all the written content of the workshops, accompanied by podcast-style audio narration for asynchronous engagement.
- Multimodal activities: An online version of the workshop activities was developed. A physical space for mapping was created, which would be mirrored for online engagement using kumu.io virtual platform.
- Diverse advertising: Posters were created and visibly showcased in a dedicated space in a high-traffic area of the hospice. The posters contained all the content of the workshops, prompts, and a QR code that linked to the themes corresponding to the website page. Recruitment emails and face-to-face interactions were also used to promote participation.
- Extended flexible access: The workshop content was made available for 4 weeks, allowing participants greater flexibility, with the workshop facilitator available for drop-ins or scheduled one-to-one and group sessions.

#### Participant Recruitment and Data Collection

Textbox 2 outlines participant inclusion and exclusion criteria. Ethical approval was granted, as well as staff and patient participants were recruited through a combination of methods which evolved from iteration 1 to iteration 2. In workshop iteration 1, participants were recruited through advertising posters exhibited in the hospice, emails, and face-to-face interactions. The same-time daily format was expected to build momentum among those who attended. However, the passive advertising approach to engagement, that is, posters and emails proved ineffective, although it raised wider awareness of the workshops among hospice staff and patients.

In workshop iteration 2, a more direct yet flexible recruitment strategy was adopted, considering potential participants already had some level of awareness about the content and remit of the workshops through iteration 1 recruitment activities. For patient recruitment, staff were briefed to identify receptive groups and introduce the project after their regular activities, allowing interested patient participants to engage without disrupting their planned medical and care routines. For staff recruitment, the researcher coordinated directly or through line managers to schedule group or individual sessions, offering rescheduling options when needed.

Textbox 2. **Participant inclusion and exclusion criteria.**

**Inclusion**

- A person who has had experiences with the hospice, including, but not limited to, patients, family members of current or past patients, staff, and volunteers
- For patient participants, a gatekeeper or ward sister team must confirm that the individual is willing and able to consent and participate in the workshops
- Participants must be able to provide informed consent independently
- Participants must be 18 years of age or older
- Participants must be able to communicate effectively in English to engage in workshop discussions
- Participants must be willing and able to commit to the time required for workshop participation

**Exclusion**

- A person who has limited or no interaction with the hospice, including, but not limited to, mail delivery person, third-party contractor
- For patient participants, if a gatekeeper declares that a person is unable to consent to being a part of the study or is too vulnerable to participate
- Persons unable to provide informed consent independently, as determined by a designated gatekeeper or ward sister
- Individuals under 18 years of age
- Individuals with cognitive impairments that would prevent them from meaningfully engaging in the workshop activities
- Individuals who cannot communicate effectively in English to engage in workshop discussions

## Results

### Participant Characteristics and Overview of the Hospice System Map

**Figure 1.**
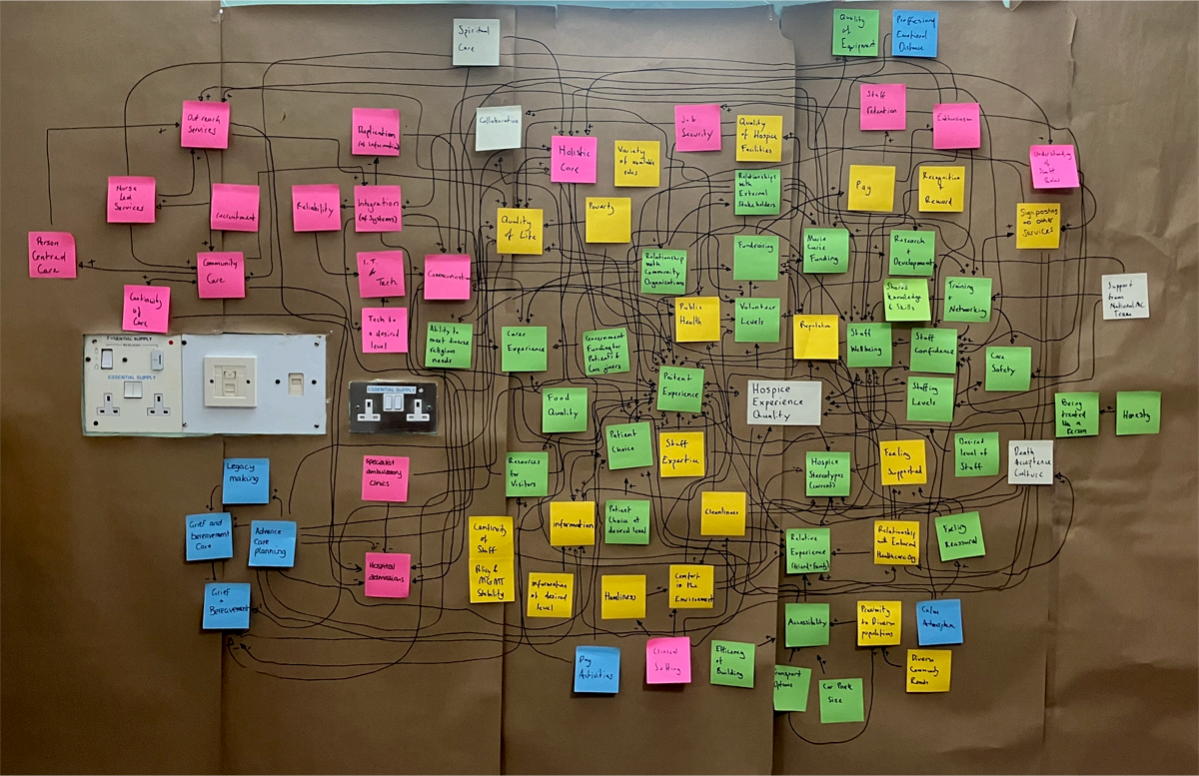
The physical causal loop diagrams (CLD) codeveloped by hospice stakeholders as system mapping workshop participants.

**Figure 2.**
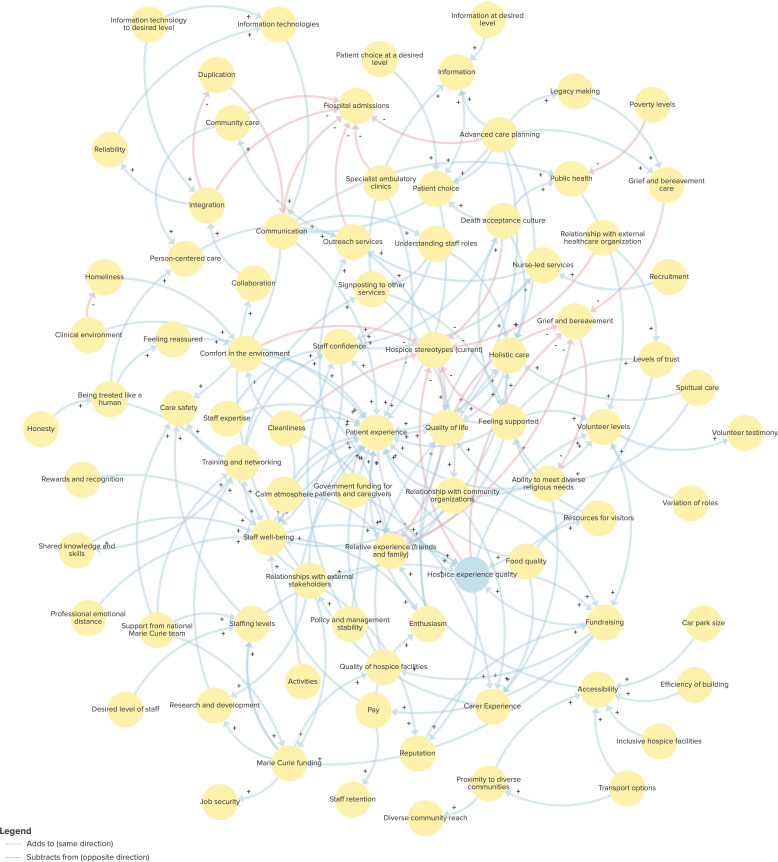
The online version of the participatory causal loop diagrams (CLD).

Overall, 27 participants representing 12 diverse and distinct hospice stakeholder groups attended the workshops. The participating stakeholder groups included patients, doctors, health care assistants and nurses, community nurses, volunteers, management, chaplaincy, well-being team, therapy team, research team, maintenance team, and patient and family support team. This was considered a highly representative sample of hospice care stakeholders. Figures 1 and 2 represent the physical and digital versions of the participatory hospice system map, respectively, codeveloped by hospice stakeholders as workshop participants. The system map contains 84 variables with 175 connections and a network density of 0.03, meaning that the network contains 3% of the possible edges (connections) expected in a completely interconnected network, indicating a relatively sparse network structure compared with a fully connected system.

The participatory and iterative mapping process meant that the hospice system map was validated iteratively and incrementally through each round of stakeholder participation. Participants were asked to both add to the map and check whether the existing variables and their connections on the map were correct, complete, and well explained according to their stakeholder view of the hospice system. In the later stages of map codevelopment, a saturation point was reached where participants describing the effect of a variable could find that connection already represented on the map.

CLDs offer the potential for mixed-method analysis, contributing to a composite understanding of complex systems both quantitatively and qualitatively. The findings presented here are preliminary and represent the outcomes of this process rather than fully articulated results. Further review for missed connections and redundancies, alongside triangulation with other methods or literature, is required for a more rigorous causal understanding of the system. Quantitative and qualitative findings at this stage are presented using network analysis and qualitative interpretation.

### Network Analysis Findings: Key Variables and Causal Interrelationships in the Hospice System According to Participatory Stakeholder Mapping

Network analysis enables the quantification of the structure of a network and provides insights into the influence and importance of variables on and within the structure, as well as the flow of information within the network [47]. Four commonly used metrics in network analysis, that is, in-degree, out-degree, betweenness centrality, and closeness centrality, were captured.

**Figure 3.**
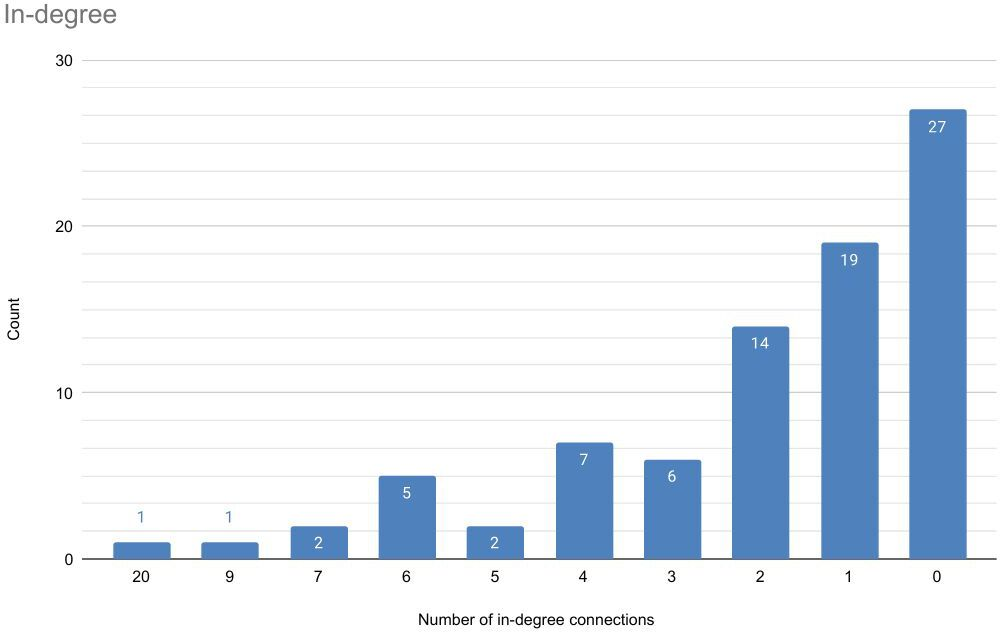
Number of in-degree connections.

**Figure 4.**
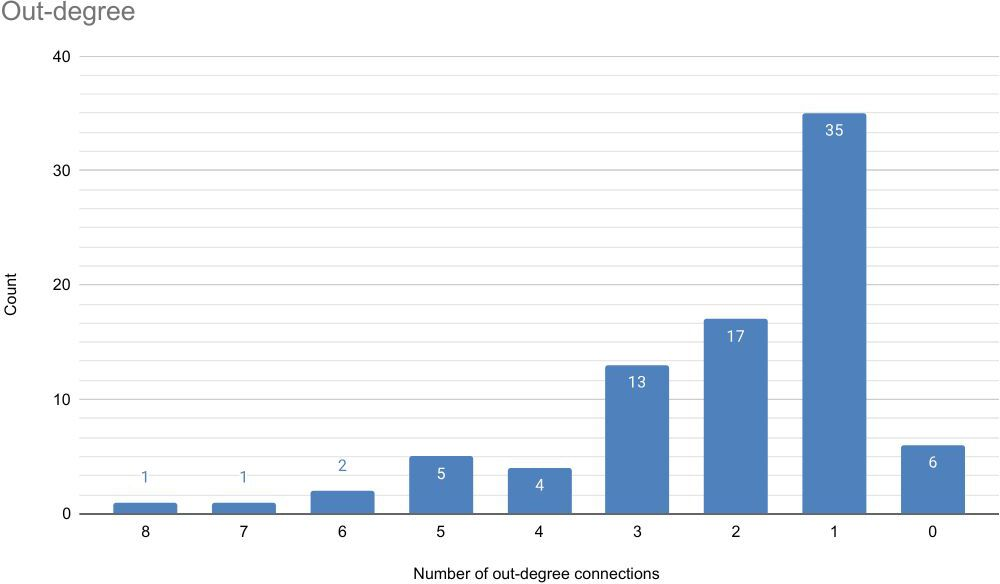
Number of out-degree connections.

In-degree measures the number of incoming connections for a variable. Variables with high in-degrees are impacted by multiple other variables. An in-degree of 0 means a variable is not impacted by anything in the system. Out-degree measures the number of outgoing connections for a variable. High out-degree variables can influence more in the system. Variables with a 0 out-degree will not directly impact the system. The codeveloped hospice system map demonstrated in-degree variables ranging from 20 to 0 (Figure 3) and out-degree variables ranging from 8 to 0 (Figure 4).

Betweenness centrality measures a variable’s frequency on the shortest path between 2 other variables. Variables with high betweenness have more control over the flow of information and act as key bridges within the network, referred to as “Mediators” [47]. Closeness centrality measures the distance each variable is from all other variables. In general, variables with high closeness can spread information to the rest of the network most easily and usually have high visibility into what is happening across the network [48]. However, centrality measurements are to be viewed critically. As pointed out by Crielaard et al [49], centrality has been found to vary in results with an adjustment of the positioning of variables in mental models especially as it is a directionless measurement in a directioned map. Table 2 presents the top 3 variables with the highest out-degree, in-degree, betweenness centrality, and closeness centrality out of 84 variables identified. The complete table providing full details is available in Multimedia Appendix 4.

**Table 2.** Network analysis results according to out-degree, in-degree, betweenness centrality, and closeness centrality.

Variable	Out-degree	In-degree	Betweenness	Closeness
Top 3 out-degree				
Advanced care planning	8	0	0.00	0.22
Feeling supported	7	2	0.04	0.21
Relationships with external stakeholders	6	2	0.13	0.23
Relationship with community organizations	6	1	0.02	0.23
Top 3 in-degree				
Patient experience	1	20	0.11	0.14
Staff well-being	4	9	0.08	0.18
Relative experience (friends+family)	2	7	0.08	0.15
Staff confidence	1	7	0.03	0.13
Top 3 betweenness				
Fundraising	4	4	0.19	0.20
Ability to meet diverse religious needs	3	2	0.14	0.17
Grief and bereavement	1	4	0.14	0.13
Relationships with external stakeholders	6	2	0.13	0.23
Top 3 closeness				
Relationships with external stakeholders	6	2	0.13	0.23
Relationship with community organizations	6	1	0.02	0.23
Advanced care planning	8	0	0.00	0.22
Signposting to other services	3	2	0.09	0.21
Feeling supported	7	2	0.04	0.21

According to the network analysis of CLDs, a key variable is considered one that has a high degree of connectivity, influences multiple other variables, or bridges different parts of the system and, therefore, is identified using the “in-degree,” “out-degree,” “closeness,” and “betweenness” measures. Key causal interrelationships are considered ones that form feedback loops, connect otherwise disparate parts of the system (*betweenness*), or have a significant influence on the system’s behavior over time.

A leverage point is defined as a variable or relationship in the system where a small change could lead to large shifts in the system’s behavior. According to network analysis, these include the *highest out-degree, highest closeness, and highest betweenness* [47]. *Highest out-degree* affects a lot in a system; therefore, changing it should see an effect on many elements quickly. The highest betweenness signifies a bottleneck between clusters, and changing it should see greater connections between variables in the system. The highest closeness affects and reflects the health of the whole system, and changes in it should see a reciprocal effect across the system. Table 3 outlines key variables, causal interrelationships (RQ1), and leverage points (RQ2) in the hospice care system as identified through participatory system mapping with stakeholders.

**Table 3.** Identified key variables, causal interrelationships, and leverage points in the hospice system.

Variables	Variables, causal interrelationships, and leverage points
Key variables and causal interrelationships	
Highest in-degree	Patient experience (20)
Highest out-degree	ACP^a^ (8)
Highest betweenness	Fundraising (0.19)
Highest closeness	Relationships with community organizations (0.23); relationships with external stakeholders (0.23)
Highest number of subtractive connections	Hospice stereotypes (6); hospital admissions (6)
Key leverage points	
Highest out-degree	ACP
Highest betweenness	Fundraising
Highest closeness	Relationships with community organizations and relationships with external stakeholders
A mindset variable that affects many stakeholder variables	Feeling supported
A mindset to how we view the role of stakeholders in a system	Stakeholder terminology

a ACP: advanced care planning.

### Qualitative Findings: Systemic Support and Role Perception

#### Limitations of Centrality Measures

While network analysis can provide valuable quantitative insights, it has its limitations. Using betweenness and closeness centrality measures to identify leverage points can lead to incorrect inferences [49]. Furthermore, higher leverage points, such as mental models as described by Meadows [50], would not be shown as a single or group of variables within a CLD system map but are identified through an understanding of the whole. Understanding these limitations and informed by the results from network analysis, the discussion section qualitatively and critically examines the system through a lens of stakeholder values, discovering key variables, and their influence and importance within the system.

#### Feeling Supported

“Feeling supported” emerged as a highly connected variable in the hospice care system, with positive effects on patient and relative experiences, staff confidence, volunteer levels, levels of trust, and death acceptance culture, while also reducing hospice stereotypes. The map does not show how each stakeholder group experienced support, especially where patients’ and staff’s understandings may differ, so this remains an area for future focused interrogation and causal analysis.

The broader significance of this variable is that it points to a more holistic role for hospices, not only in relation to patients but across all stakeholders. If the aim is to help people feel supported beyond treatment, cure, or care for them, the emphasis shifts toward a more collaborative and autonomous interaction rather than a one-directional staff-to-patient action. This is not to suggest we change the terminology of care but to explore alternative mindsets for how PEoLC service systems enact care and for whom. This also aligns with the discussion by Sawyer et al [51] of palliative care as a “shared social process,” in which death, dying, and loss are shaped through relationships between services, communities, and individuals. The ethics of care proposed by Tronto [52] offers a further relational lens, as it foregrounds care as a set of relationships shaped by power, dependency, and responsiveness, and may help frame this emergent leverage point for further analysis.

#### Stakeholder Characteristics

Analysis of how participants described stakeholder variables revealed distinct mindsets that highlight potential system leverage points and areas for further research. Patients, families, and carers were associated with “experiences,” staff with “well-being,” volunteers with “levels,” and organizations and community stakeholders with “relationships.” These differences reflect implicit assumptions about the roles and values assigned to each stakeholder group within the hospice care system.

Examining the values and mindsets attached to these variables may uncover underrecognized contributions and support a re-evaluation of stakeholder roles. For example, reframing volunteers through the lens of “experience” rather than “levels,” as suggested by the NHS Volunteering Taskforce [53], could alter how their role is perceived and designed within the system. This shift could move volunteers from being viewed mainly as a source of labor toward being seen as people with whom relationships are actively built.

This does not require changing the terminology of care itself, but it does suggest the value of extending person-centered principles to other stakeholders. In that sense, the variable names themselves point to a broader reimagining of power dynamics and responsibilities across the hospice system.

## Discussion

### Key Variables and Causal Interrelationships in the Hospice System (RQ1)

Key variables and their causal relationships, according to the CLD network analysis metrics, are discussed below.

#### Patient Experience (Highest In-Degree)

Patient experience had the highest in-degree connections (20), showing that the system focuses on improving it, with its sole out-degree connection to central hospice experience quality. Stakeholders mapped hospice experience quality as influencing hospice stereotypes, grief or bereavement, reputation, fundraising, and volunteer levels. Negative public perceptions (hospices as a place of death and sadness) persist, but positive patient experiences challenge these, motivating volunteering and fundraising among direct beneficiaries, though broader outreach is needed to reshape stereotypes beyond service users.

#### Advanced Care Planning (Highest Out-Degree)

Advanced care planning (ACP) showed the second-highest out-degree connections (8), reaching 37% of system variables and ranking third in closeness centrality. It linked positively to quality of life or holistic care and negatively to hospital admissions, aligning with the findings of Brinkman-Stoppelenburg et al [54] that ACP improves end-of-life care, increases hospice use, and reduces hospitalizations.

Critically, ACP had no in-degree connections, revealing stakeholder uncertainty about its implementation drivers, echoing the documented cultural and operational barriers reported by Lund et al [55] and Jimenez et al [56] despite recognized benefits. This gap underscores the need for reexamining ACP aims, delivery, and suitability within hospice settings.

#### Fundraising and Relationships With Community Organizations and External Stakeholders (Highest Centrality Outcomes—Betweenness and Closeness)

Fundraising ranked highest for betweenness centrality (0.19, excluding the central variable), indicating a system bottleneck and a single point of failure. This is corroborated by UK Parliament evidence calling current PEoLC funding “not fit for purpose,” requiring commissioning reform [57].

Relationships with community organizations (1st closeness, 0.23) and external stakeholders (2nd) suggest a broad reach, though site-specific. Findings align with the literature on hospice-nursing home collaborations, hospice-university networks, clinician-researcher partnerships, and compassionate communities building resilient PEoLC networks [7,58-60].

#### Hospice Stereotypes and Hospital Admissions (Highest Variables With Subtractive Interrelationships)

Stakeholders mapped hospice stereotypes and hospital admissions for reduction (combined 12 negative in-degrees, 1 out-degree), showing clear drivers but limited understanding of downstream effects—risking the perpetuation of existing behaviors.

Review by Robinson et al [39] confirms hospitals’ poor PEoLC fit: high symptom burden, poor communication impairing family decisions, noisy and busy environments despite occasional dignified care.

### Leverage Points in the Hospice System (RQ2)

#### Structural and Conceptual Leverage Points

Leverage points are system locations where small shifts yield significant changes. Meadows [50] hierarchy ranges from shallow (parameters) to deep (paradigms) interventions.

Network analysis identified structural leverage through the *highest out-degree, closeness*, and *betweenness centralities*. Complementary qualitative analysis revealed conceptual leverage points, combining context-agnostic metrics with stakeholder-specific insights for a comprehensive understanding of the hospice system and intervention opportunities.

#### Fundraising

Network analysis identified fundraising as leverage across Meadows hierarchy. Shallow interventions could expand activities and sources to increase fundraising, while deep paradigm shifts might diversify income beyond the current UK hybrid NHS and charity model. Recent parliamentary calls and PEoLC manifestos recognize funding pressures [3,61], aligning with the Health and Care Act 2022 [62] commissioning mandate and palliative care’s human rights framing, making full government funding a compelling ethical and systemic solution.

#### Relationships With Community Organizations and External Stakeholders

The importance of relationships with community organizations and external stakeholders suggests that greater integration of care across NHS services and into the community could strengthen the wider PEoLC system. Public health and community models of palliative care aim to improve end-of-life experiences by combining palliative principles with public health strategies and community services. These approaches recognize that care for people with life-limiting illnesses extends beyond traditional health care, and existing evidence suggests benefits including more deaths at home, fewer emergency department presentations, higher patient and carer satisfaction, shorter hospital stays, and fewer admissions and readmissions [51,63,64].

#### Advanced Care Planning

There are multiple levels at which ACP could be leveraged. At a practical level, this could include better identification of patients, earlier documentation, and expanding who initiates and records ACP beyond medical professionals. At a deeper level, it could involve a mindset shift, reframing ACP as “Advanced Care Preparation” so that it is understood as preparation for end-of-life care rather than as a fixed planning exercise [65]. Beyond this, Morrison et al [66] argue that ACP may not be the best model at all if it continues to fail its intended aims, and that alternative ways of communicating current, rather than future, end-of-life care plans may be more valuable.

Beyond the numerical analysis and the more formulaic, context-agnostic use of network analysis, other factors, such as the number of stakeholder links and the characteristics of each variable, were also considered. This helped identify 2 further leverage points in the hospice system map.

### Methods for Mapping Hospice Systems (RQ3)

Participatory system mapping, as part of a design workshop series with hospice stakeholders, proved to be an effective method overall for capturing complex system dynamics in a hospice setting. The research resulted in the iterative development of a hybrid multimodal extended asynchronous method, which improved interaction with participants; accommodated a range of participant preferences in engagement style and digital literacy levels; and allowed for spatiotemporal barriers to be elevated to a degree. The method’s strengths lie in its ability to engage diverse stakeholders, capture in-depth perspectives, and adapt to the unique constraints of the hospice environment. However, challenges related to time constraints, engagement with complex visual data, and the need for researcher guidance in novel tasks suggest areas for refinement or further examination. Future applications should consider a balanced approach between face-to-face and online interactions, with careful attention to spatial and temporal factors unique to each hospice setting. Multimedia Appendix 5 outlines the key strengths and challenges of hybrid asynchronous extended design workshops as a method for participatory system mapping with stakeholders in a hospice setting.

### Suitability of Data Collection Method

Participatory system mapping offers advantages and disadvantages when compared with alternative systemic mapping methods such as interviews [67], GMB [68], and rich picture mapping [69]. While interviews may provide greater depth into individual perspectives, participatory system mapping supports a collective understanding using participants’ own language, with ideas refined through an ongoing cocreation process rather than filtered solely through researcher interpretation, making it useful as a foundation for continued participatory development, such as co-design. Unlike traditional GMB, the asynchronous approach may increase overall engagement, although it may sacrifice some of the insights generated through real-time group discussion. Rich picture mapping differs from CLD by offering a more open and expressive format, but it may provide less direct structure for systemic analysis.

While the network analysis does not directly demonstrate how to improve patient outcomes, it helps identify where communication, coordination, support, and resource pathways may be strengthened within the hospice system, offering a starting point for more holistic and participatory service investigation and development in PEoLC. Overall, participatory system mapping using CLD provides a balance between accessibility, collective insight, and structured representation of system dynamics, making it well suited to system mapping in sensitive health care settings. Multimedia Appendix 6 summarizes the strengths and weaknesses of the method used in this study compared with alternative approaches.

### Limitations and Future Work

CLD-based system mapping has inherent capabilities and limitations in terms of the information it can provide on the what, how, and why of a system’s variables and interrelations. In terms of generalizability and thoroughness, this participatory system map provides a snapshot of a single hospice system in an urban context in Northwest England and is solely defined based on the existing understanding and implicit assumptions of its participating stakeholders. Future work could attempt research triangulation by mapping the same system through distinct complementary methods, for example, observations and interviews, thereby enhancing validity and advancing methodological granularity and comparison.

In terms of representativeness and completeness, all identified hospice stakeholder groups were successfully engaged in codevelopment of the system map, apart from the patient family members who could not engage in workshops due to multiple factors. The qualitative interpretation of a CLD is inherently subjective and could be influenced by researcher bias; hence, the combination of quantitative and qualitative CLD analysis in this study.

A CLD system map acts as an analytical canvas, enabling open-ended, multivariable, and granular analysis and interpretation of the whole or parts of a system, depending on the questions being asked about that system. This paper explored key variables, interrelationships, and leverage points of a hospice system as a first step, laying the foundations for the use of CLD in understanding complex hospice system dynamics, and further question-specific discussion of findings could be included.

A greater understanding of the importance of variables and connections could be discovered using Barbrook-Johnson and Penn’s [33] suggestion of weighted connections. However, this would introduce greater complexity and time demands for the workshop activities and facilitation, and it should be approached as a trade-off. The researcher-participant dynamic, through the facilitation of the workshops, may have positively influenced the willingness of participants to share sensitive information, with a nonmedical researcher who lacks the authority to implement changes to the organization directly.

### Conclusions

This study developed an innovative hybrid, asynchronous multimodal design workshop to engage 27 diverse hospice stakeholders in participatory system mapping, creating a composite representation of a hospice care system consisting of 84 variables with 175 connections. Key methodological contributions include a reflexive and responsive participatory approach and method that was mostly successful in overcoming challenges common to researching in PEoLC environments, thereby developing a potentially replicable model for ethically engaging diverse stakeholders.

Empirical contributions, derived through mixed-method analysis, reveal structural leverage points discovered in funding, a system bottleneck, and the importance of community organizations and external stakeholder relationships to the system. Further conceptual leverage points identified include reimagining stakeholder roles and expanding the principle of “feeling supported” holistically to the stakeholders of the system. These findings reflect current initiatives found in literature, health care practice, and policy [3,7,53,57,62]. These findings reflect sector-wide challenges and developments reflected from a single site; however, with an absence of family member participants, we lack a critical perspective for a more holistic understanding.

In conclusion, this research provides an informed framework for future participatory studies in PEoLC environments and potentially in broader health care contexts. It demonstrates how accessible participatory methods can produce a holistic understanding of complex systems, generating actionable and grounded insights. Future research could explore the replicability and application of this approach across diverse health care settings aiming to create stakeholder-informed system changes.

## Supplementary material

10.2196/69746 Multimedia Appendix 1 An overview of expansion of palliative and end-of-life care.

10.2196/69746 Multimedia Appendix 2 Study protocol describing the study design, participant recruitment, consent procedures, and eligibility criteria.

10.2196/69746 Multimedia Appendix 3 Ethics acceptance letter.

10.2196/69746 Multimedia Appendix 4 Full network analysis results for, out-degree, in-degree, betweenness centrality, and closeness centrality.

10.2196/69746 Multimedia Appendix 5 Key strengths and challenges of the participatory system mapping workshop method.

10.2196/69746 Multimedia Appendix 6 Strengths and weaknesses of the participatory system mapping workshop method compared to alternative mapping methods.

## Abbreviations

ACP: advanced care planning
CLD: causal loop diagram
GMB: group model building
NHS: National Health Service
PEoLC: palliative and end-of-life care
