# Supplementary material for: Participatory System Mapping of a Hospice Care System With Hospice Stakeholders: Hybrid Design Workshop Study
Source: J Particip Med. 2026 Jul 24;18:e69746. doi: 10.2196/69746 (PMC13399969; doi:10.2196/69746)
Supplement: Multimedia Appendix 2 [file jopm-v18-e69746-s002.docx]

## STUDY PLAN

Four methods will be used in this study that aims to give a broad foundation of current practice and perspectives on future service requirements. We will be conducting an observational study, semi-structured open-ended interviews, a survey and workshops.

### ORDER AND TYPE OF STUDY METHODS

1. First, an observational study will be conducted to map the interactions within hospice care objectively. This will help to map the various connections between humans, objects and space.

2. Secondly, semi-structured, open-ended qualitative interviews will be conducted with hospice patients, hospice patients' families, and staff and third-party affiliates. This format will allow keeping the style conversational and exploratory. This format should also allow for exploration and for flexibility in working with patients, visitors and staff's changing work schedules. Questions will focus on the experience of hospice care, what have been the positive interactions, what has been necessary and what has been challenging or acted as barriers to better care, also perspectives on where future hospice care is currently heading and where it needs to be heading (aimed at staff specifically)?

3. Thirdly, a survey will be used to establish value priorities of the hospice care service.

4. Finally, a co-define and co-design workshop with diverse hospice stakeholder groups including patients, family, staff and third parties will be held to establish a range of future hospice care scenarios which are both human-centred and technologically advanced. This group of participants will form a ‘Lead Design Forum’, which will be instrumental throughout the project and in future applications as an advanced PPI element.

### CONTEXT AND ADOPTED APPROACH

In order to ensure the study is conducted in the most contextual, integrated and ethically appropriate way, and as part of a longer-term partnership between the research team and Marie Curie Hospice, we will adopt a 'designer-in-residence' research approach where the design researchers will be embedded into the hospice building and present on-site as honorary researchers appointed by Marie Curie Hospice. The PhD researcher and their supervisor will have formal 'Honorary Researcher' contracts with Marie Curie Hospice and all research activities (observation, interview, survey and workshop) conducted will be overseen by the research team at Marie Curie Hospice, in particular the Director of Research.

### OVERSIGHT AND MANAGEMENT OF RESEARCH

The Director of Research at Marie Curie Hospice will act in a 'line manager' capacity, ensuring research integrity, researcher and participant capacity and well-being and ethical conduct. In addition to this, the researchers will receive communication skills training specific to researching within the environment - considering the context, patients and visitors, and interactions specific to a hospice environment.

### RECRUITMENT OF PARTICIPANTS AND GAINING CONSENT

**1. Observational study**

1.A - Hospice staff, both medical and non-medical; Through an introduction to the hospice at the beginning of the residency to allow staff time to read over the information and return consent forms. For staff who may not be present in the hospice during the introductory stage, the Director of Research at Marie Curie will disseminate the information sheets and consent forms with the researcher's email address electronically to staff members as news items.

1.B - Patients: The eligibility of patients to participate in any research will be discussed and assessed daily as part of a 'huddle' meeting which is held every morning at the Hospice by the MDT (Multidisciplinary Team) and is used as an assessment of patient condition and wellbeing. Criteria of participation eligibility will include; an appropriate level of English language speaking, mental capacity, physical capacity, infection control, level of stability of patient's condition, irritability, and consciousness. If there is a chance that the patient's mental or physical capacity has deteriorated during the day, the research will be stopped and await assessment from the Multidisciplinary Team meeting the following day. If the patient with whom the research activity is not fully concluded yet, has been assessed as unable to give informed consent any further, the data gathered the previous day/s will be destroyed and no further research will take place with that patient.

1.C - Patients' families: When observing eligible and consenting patients who are visited by family members the researchers will cease observations of the patient that day, inform the family of what we are doing and if they would be interested in participating in their subsequent visits. If so we will provide an information sheet and consent form to be read over and the consent form returned to the researchers in the potential participants' own time. If consent is not given, we will not observe the family members' interactions.

1.D - Third parties and affiliates: During the observational study researchers will be alert to third parties and affiliates that physically interact with the Hospice. The researchers will introduce the topic of research, when and if appropriate to do so (e.g. they are not performing a work task) and firstly enquire if they were likely to be regularly returning to the hospice in this capacity and if so if they would consider participating in the observational study. If it is likely to be a different member of staff each time we will enquire if information sheets and consent forms could be disseminated within the company through them and await responses. On-site verbal confirmation of identity and completed consent forms will be necessary to observe these participants.

**2. Interviews**

2.A - Hospice staff, both medical and non-medical; Interview participants will be recruited on-site, either by volunteering to participate with prior engagement with the study or designer in residence, or by responding to an email advert disseminated by the Director of Research, or by responding to an on-site poster detailing the study and an email address and contact number to respond to, if interested.

Interested participants will be emailed with the study information reiterated, for clarity (please see email advert) and consent forms will also be included in the email sent. The researcher's email will be provided on the information sheets sent in the original email for participants to get in touch if any more questions about the study are necessary. Additionally, we offer a quick introductory conversation, to provide more background on the research and answer any possible questions for any participants prior to them deciding whether to participate in the research or not.

2.B - Patients: Firstly, patients must be assessed as eligible by a multidisciplinary team as part of their routine daily meetings to assess ongoing healthcare requirements and status. If eligible we will seek an introduction from a qualified staff member, where we can outline the study and provide a physical or electronic information sheet along with a consent form that the patient can consider and respond to in their own time.

2.C - Patients’ Family: Patients' families will be recruited from either prior engagement with the study or designer in residence, from a connection to a patient who has consented to be interviewed and has recommended their family to participate, or from an on-site poster detailing the study and an email address and contact number respond to, if interested.

2.D - Third parties and affiliates: Interview participants will be recruited either by volunteering to participate with prior engagement with the study or designer in residence or by responding to an on-site poster detailing the study and an email address and contact number to respond to, if interested. We will enquire from Marie Curie staff if there are other stakeholders in this group that would not physically interact with the Hospice that would be valuable to contact, in which case we shall enquire to those third parties' general enquiries team about the study and how we may contact potential participants ethically.

**3. Survey**

3.A - Hospice staff, both medical and non-medical;

3.B - Patients and their families: Family; will primarily be recruited from patients who have already expressed an interest in participating in the study and would be happy to pass on the information and consent forms to their families. Other forms of recruitment will be through a hospice community board advert with the researcher's email address attached to request further information and consent.

3.C - Third parties and affiliates: Will primarily be recruited from prior engagement to the study. Other third-party recruitment will come from the recommendation of Marie Curie, in this case, we will pursue recruitment through a public domain email of that third-party

**4. Workshops**

Workshops will be recruited from the pool of interview participants (patients, family, staff and third parties) . We will offer them to take part in the next stage of research as 'forum members' if they are interested we will provide information sheets and consent forms for them to consider and respond to.

We will host two workshops within the Marie Curie Liverpool building, with permission and coordination from Marie Curie to allow staff the time to participate and to provide a support nurse for all participants if required. Immediately before the workshop begins we will verbally confirm that everyone attending the workshop has given consent.

Patients will have to be considered eligible on the day of the workshops by the MDT to be able to continue to give consent.

### ELIGIBILITY AND CONTINUED PARTICIPATION OF PATIENT PARTICIPANTS

The eligibility of patients to participate in any of the four research methods listed above will be discussed and assessed daily as part of a 'huddle' meeting which is held every morning at the Hospice by the MDT (Multi-Disciplinary Team) and is used as an assessment of patient condition and wellbeing. Criteria of patient participation eligibility will include; level of English language speaking, mental capacity, physical capacity, infection control, level of stability of patient's condition, irritability, and consciousness.

If there is a chance that the patient's mental capacity has deteriorated during the day, the research will be stopped and await assessment from the Multidisciplinary Team meeting the following day. If the patient with whom the research activity is not fully concluded yet, has been assessed as unable to give informed consent any further, the data gathered the previous day/s will be destroyed and no further research will take place with that patient.
