# Supplementary material for: Participatory System Mapping of a Hospice Care System With Hospice Stakeholders: Hybrid Design Workshop Study
Source: J Particip Med. 2026 Jul 24;18:e69746. doi: 10.2196/69746 (PMC13399969; doi:10.2196/69746)
Supplement: Multimedia Appendix 3 [file jopm-v18-e69746-s003.pdf]

UNIVERSITY OF LIVERPOOL

Central University Research Ethics Committee B

27 May 2022

Dear Dr Nickpour

I am pleased to inform you that your application for research ethics approval has been approved. Application details and conditions of approval can be found below. Appendix A contains a list of documents approved by the Committee.

Application Details

Reference:	10901
Project Title:	Co-designing the future of hospice care
Principal Investigator/Supervisor:	Dr Farnaz Nickpour
Co-Investigator(s):	Mr Andrew Tibbles
Lead Student Investigator:	-
Department:	Civil Engineering and Industrial Design
Approval Date:	27/05/2022
Approval Expiry Date:	Five years from the approval date listed above

The application was **APPROVED** subject to the following conditions:

Conditions of approval

Please note: Any research ethics approval granted will be subject to the University's Policies on research during the pandemic.

Please ensure you are familiar with the latest guidance on conducting research during the pandemic. The guidance is available on the research ethics webpages.

- All serious adverse events must be reported to the Committee (ethics@liverpool.ac.uk) in accordance with the procedure for reporting adverse events.
- If you wish to extend the duration of the study beyond the research ethics approval expiry date listed above, a new application should be submitted.
- If you wish to make an amendment to the study, please create and submit an amendment form using the research ethics system.
- If the named Principal Investigator or Supervisor changes, or leaves the employment of the University during the course of this approval, the approval will lapse. Therefore it will be necessary to create and submit an amendment form within the research ethics system.
- It is the responsibility of the Principal Investigator/Supervisor to inform all the investigators of the terms of the approval.

Kind regards,

Central University Research Ethics Committee B

ethics@liverpool.ac.uk

CUREC-B

Appendix - Approved Documents

(Relevant only to amendments involving changes to the study documentation)

The final document set reviewed and approved by the committee is listed below:

Document Type	File Name	Date	Version
Questionnaire	Questionnaire (2)	29/12/2021	1
Participant Information Sheet	Patient & Family Observational - Participant Information Sheet.docx	11/05/2022	1.1
Participant Information Sheet	Staff Observational - Participant Information Sheet.docx	11/05/2022	1.1
Participant Consent Form	Observational Participant Consent Form.docx	11/05/2022	1.1
Advertisement	Interview Poster Advert.docx	12/05/2022	1
Research Tools	Off,Campus,Risk,Assessment,Form	12/05/2022	1
Interview Schedule	Patient _ Family interview schedule_V1.1	13/05/2022	1.1
Interview Schedule	Staff interview schedule_V1.1	13/05/2022	1.1
Advertisement	Interview Email invitation	13/05/2022	1.1
Participant Information Sheet	Interview Participant Information Sheet	13/05/2022	1.1
Participant Consent Form	Interview Participant-Consent-Form	13/05/2022	1.1
Debriefing Material	Interview Debrief	13/05/2022	1.1
Participant Information Sheet	Questionnaire Participant Information Sheet	13/05/2022	1.1
Participant Consent Form	Questionnaire Participant-Consent-Form	13/05/2022	1.1
Advertisement	Workshop Email-invitation	13/05/2022	1.1
Participant Consent Form	Workshop Participant-Consent-Form	13/05/2022	1.1
Participant Information Sheet	Workshop Participant Information Sheet	13/05/2022	1.1
Study Proposal/Protocol	Study Plan 24_05	24/05/2022	1
Advertisement	Observation Poster Advert	24/05/2022	1
